# Endometrial cancer in a woman undergoing hysteroscopy for recurrent IVF failure

**DOI:** 10.1186/s10397-017-1009-1

**Published:** 2017-04-25

**Authors:** Pietro Gambadauro, Johannes Gudmundsson

**Affiliations:** 1 Centre for Reproduction, Uppsala University Hospital, 751 85 Uppsala, Sweden; 2 Karolinska Institute, LIME/NASP-C7, 17177 Stockholm, Sweden; 3 Department of Women’s and Children’s Health, Uppsala University, 751 85 Uppsala, Sweden

**Keywords:** Embryo transfer, Endometrial cancer, Hysteroscopy, Implantation failure, In vitro fertilization

## Abstract

**Background:**

Hysteroscopy, despite being the undisputed gold standard for the examination of the uterine cavity, is controversial as a routine procedure in infertile women. However, benign intrauterine conditions are common in women suffering repeated in vitro fertilization (IVF) failure, and growing evidence suggests a unique diagnostic and therapeutic role for hysteroscopy. Endometrial malignancy, on the contrary, is unreported by large published series of women with repeated IVF failures undergoing hysteroscopy, and its impact on fertility, for obvious reasons, has not been studied.

**Results:**

An unsuspected endometrial cancer was diagnosed in an asymptomatic 38-year-old woman undergoing hysteroscopy because of several repeated failures of in vitro fertilization and embryo transfer.

**Conclusions:**

Endometrial cancer can be found at hysteroscopy in young women with repeated IVF failures. The possibility of repeatedly unsuccessful fertility treatments should be taken into account when counseling infertile women about conservative treatment of endometrial cancer.

## Background

During the last decades, developments in ultrasound diagnostics and increased knowledge about the determinants of assisted reproduction’s success have caused a downgrading of gynecological endoscopy’s role in the assessment of female infertility. Hysteroscopy, for instance, in spite of being the undisputed gold standard for the examination of the uterine cavity, is controversial as a routine procedure [1]. However, growing evidence suggests a unique diagnostic and therapeutic role for hysteroscopy, especially in cases of repeated failures of assisted reproductive technology [2]. In such cases, abnormal hysteroscopic findings, such as endometrial polyps, submucous fibroids, adhesions, and septa, are common [3–5], and hysteroscopy offers an opportunity for diagnosis and a convenient see-and-treat management [2, 6]. Endometrial malignancy, on the contrary, is unreported in large published series [3–5], and its impact on fertility, for obvious reasons, has not been studied.

We here present and discuss a case of unsuspected endometrial cancer which was accidentally diagnosed in a woman undergoing hysteroscopy because of repeated failure of in vitro fertilization (IVF) and embryo transfer (ET).

## Methods

The data of this case report was obtained through retrospective chart review.

## Results

A 38-year-old woman and her male partner had been under our care for primary infertility, at the Centre for Reproduction of Uppsala University Hospital, for 3 years. She had a normal body mass index (BMI; 22 kg/m^2^) and regular ovulatory menstrual cycles. Previously, she had used combined oral contraceptives followed by an intrauterine device for 10 years. Baseline infertility investigations, including hormonal assessments for TSH and prolactin, pelvic ultrasonography, and semen analysis, were unremarkable. Tubal perviousness and no abnormalities were seen at hysterosalpingo-contrast sonography.

After the diagnosis of unexplained infertility, she had undergone three ovarian stimulations, one with clomiphene citrate, and the following two with low-dose follicle-stimulating hormone (FSH) followed by intrauterine insemination. No pregnancy had been obtained. The couple had then undergone two IVF treatments after conventional controlled ovarian stimulation, each one leading to one fresh elective single embryo transfer (SET) and to several frozen single or double embryo transfers (DET). Overall, eight embryo transfers (two fresh SET, four frozen SET, and two frozen DET) had been performed, but no intrauterine clinical pregnancy was ever achieved. A biochemical pregnancy occurred after the third transfer of the series (frozen). The fifth ET (frozen) resulted in a tubal pregnancy, which was managed by laparoscopic salpingectomy.

Prior to the start of a new controlled ovarian stimulation for IVF-ET, it was agreed to perform a hysteroscopy to rule out intrauterine abnormalities, in view of the several previous failures. At hysteroscopy, a small polypoid growth, having its base at the fundal region, was seen. Pathology of the resected specimen returned a diagnosis of endometrial atypia. After counseling, a conservative treatment with oral progestins (medroxyprogesterone acetate 10 mg daily) was commenced. However, an outpatient endometrial biopsy by pipelle at a 3-month follow-up showed endometrial cancer of endometrioid type. The patient was thoroughly counseled by fertility and oncology specialists about the possible therapeutic strategies, ranging from conservative treatments with progestins to the standard surgical staging for endometrial cancer. As a result of her informed choice to undergo surgery, a total hysterectomy with bilateral salpingectomy and preservation of the ovaries was performed by the gynecologic oncology surgeons. Surgery and the postoperative period were uneventful. The final pathology report described a highly differentiated, diploid, endometrioid adenocarcinoma of the endometrium which was classified as FIGO stage IA (G1). No adjuvant treatment was needed. At all planned follow-up visits, in accordance with local guidelines, she was always disease-free and reported a 100% score on quality-of-life measures. At our last contact, 5 years after the hysterectomy, she also reported having adopted a child and enjoying her motherhood.

## Discussion

Hysteroscopy is not universally considered a routine procedure for the evaluation of the uterine cavity in subfertile women [1]. However, there is a high prevalence of previously undetected intrauterine abnormalities in IVF patients, particularly following to failed treatments [3–5]. This gives a pragmatic measurement of the diagnostic potential of hysteroscopy, if we consider that women with failed treatments constitute a selected population which has obviously undergone several prior ultrasound exams. Besides, growing evidence, albeit of limited quality, suggests that hysteroscopic diagnosis and, when needed, treatment may improve IVF outcomes and also be cost-effective [2, 7].

Benign hysteroscopic findings are common among IVF patients, the majority of which being represented by endometrial polyps, submucous fibroids, adhesions, or uterine anomalies [3–5]. On the contrary, an endometrial malignancy is not an expected finding in these women. Endometrial cancer, in spite of an approximate lifetime risk of 2.8% women, is a rare occurrence before 40 years old [8, 9].

Our patient was 38 years old, and no intrauterine abnormality was ever diagnosed or suspected during 3 years of repeated fertility treatments. Hysteroscopy was only performed in view of the several failures and revealed a small polypoid growth that had not been seen at ultrasound. Polyps are an increasingly common finding [3, 10]; however, their association with malignancy is controversial in younger and asymptomatic women [11]. In our case, in spite of hysteroscopic resection and oral progestins treatment, the initially diagnosed atypia turned out to be an endometrial cancer at final diagnosis, which is a known possibility [12]. The cancer was also still present on the final specimen, meaning that it was not confined to the resected polypoid area, as often reported in the literature [12]. It seems therefore worth reminding that, although conservative treatment of early stage endometrial cancer by means of progestins and hysteroscopic resection has been proposed [9, 13], the gold standard includes a total hysterectomy [14]. In this case, following a patient-centered approach to care, the choice of undergoing hysterectomy was made by the patient after thorough information about different therapeutic alternatives. In spite of that, she could still fulfill her desire for motherhood through adoption.

Whether a link existed, in this case, between infertility and the malignancy is an intriguing albeit difficult question. Infertility does not seem to represent a strong risk factor for endometrial cancer, although some conditions such as chronic anovulation in PCOS patients imply unopposed estrogenic effect on the endometrium, hence a risk for abnormal proliferation [15]. Our patient had ovulatory cycles but had undergone various ovarian stimulations with gonadotrophins as well as hormonal replacement treatments for frozen embryo transfer. Her endometrial cancer was of endometrioid type, which is closely related to estrogens. Some studies have previously shown an increased risk for endometrial cancer in women receiving gonadotrophins and clomiphene for fertility treatment although a real causal relationship is far from demonstrated [16].

One could also wonder whether the neoplasia might have played a role in the several failed treatments experienced by our patient. While benign intrauterine conditions are thought to interfere with endometrial receptivity, the hypothesis of an association of endometrial cancer with implantation failure is suggestive but unverified. This possibility should however be kept in mind when counseling subfertile patients about conservative treatments of endometrial cancer, since much of the knowledge on fertility outcomes is based on experiences with fertile women.

## Conclusions

Malignancy, albeit rare, is a possible occurrence in younger women undergoing fertility treatments. In the present case, an early diagnosis of endometrial cancer was facilitated by hysteroscopy, which was performed because of repeated IVF failures in a woman with no specific symptoms nor ultrasonographic signs of pathology. The possibility of repeatedly unsuccessful fertility treatments should be taken into account when counseling infertile women about conservative treatment of endometrial cancer.
